# Vertical jump performance is more closely associated with change-of-direction performance than 30-m average sprint speed in elite U-20 soccer players

**DOI:** 10.1371/journal.pone.0355501

**Published:** 2026-11-12

**Authors:** Arthur Placido Celestino, Robson Dias Scoz, Marcos Fernando Tiburtino, Michael Marcondes de Paula, Tobias Lima Souza, Ricardo Lima Burigo, Caio Augusto Pereira Leal, Carolina Burian Parpinelli, Walmir da Cruz, Cesar Augusto Melo-Silva, Cesar Ferreira Amorim

**Affiliations:** 1 Sport Club Corinthians Paulista, Technological Innovation Center in Sport Science, Sao Paulo, Sao Paulo, Brazil; 2 Master's and Doctoral Program in Physical Therapy, University City of Sao Paulo (UNICID), Sao Paulo, Sao Paulo, Brazil; 3 Department of Physical Therapy, College of Health Professions, The University of Texas Rio Grande Valley, Edinburg, Texas, United States of America; 4 Department of Physical Therapy, Avai Football Club, Florianopolis, Santa Catarina, Brazil; 5 Laboratory of Respiratory Physiology, School of Medicine, University of Brasilia, Brasilia, Brazil; 6 Department of Physical Therapy, Hospital of University of Brasilia, Brasilia, Brazil; 7 Department of Physical Therapy, University of Quebec, Chicoutimi, Canada; Università degli Studi di Milano: Universita degli Studi di Milano, ITALY

## Abstract

Soccer requires repeated neuromuscular actions, including jumping, sprinting, decelerating, and changing direction. This study examined the associations among vertical jump performance, 30-m average sprint speed derived from the total 30-m sprint time, and change-of-direction performance in elite U-20 soccer players. Forty-four male elite under-20 soccer players from a professional Brazilian club were assessed during the club's routine physical testing battery. Squat jump (SJ), countermovement jump (CMJ), relative power, 30-m average sprint speed, and right- and left-sided change-of-direction performance were analyzed. Pearson correlations were used as the primary analysis, with false discovery rate adjustment for multiple comparisons. Spearman correlations and partial correlations controlling for age, body mass, and height were used as sensitivity analyses. Vertical jump variables were moderately and inversely associated with change-of-direction time to both sides (r = −0.43 to −0.56), indicating that better jump performance was associated with faster directional performance. These associations remained statistically significant after false discovery rate adjustment and after controlling for age, body mass, and height. In contrast, associations between vertical jump variables and 30-m average sprint speed were weak to moderate and did not remain statistically significant. Right- and left-sided change-of-direction performances were strongly related but not interchangeable (r = 0.69), supporting bilateral assessment. These findings indicate that vertical jump performance is more closely associated with change-of-direction performance than with 30-m average sprint speed in elite U-20 soccer players. Jump testing may provide useful information about neuromuscular qualities related to directional speed tasks, but it should complement rather than replace direct change-of-direction testing.

## Introduction

Soccer is an intermittent sport that requires multiple neuromuscular actions, including running, jumping, sprinting, decelerating, and changing direction [1–6]. Many of these actions occur in decisive match situations and are frequently observed before goal-scoring opportunities [3–5]. Consequently, the assessment of neuromuscular performance is relevant for player monitoring, physical profiling, and training prescription in high-performance soccer environments.

The countermovement jump (CMJ) is widely used to monitor neuromuscular status [7–11] and fatigue during the soccer season [12]. Vertical jump performance has also been associated with other explosive performance capacities, including linear speed [13]. However, soccer performance does not depend only on straight-line sprinting. Acceleration, deceleration, and direction-specific actions are central physical demands of match play [14–18], and repeated sprint actions also contribute to competitive performance [19–23].

Previous studies have examined the relationship between vertical jump performance and speed-related outcomes in athletes. Shalfawi et al. [24] reported associations between vertical jump performance and sprint performance in professional basketball players. Köklü et al. [25] observed relationships among sprint ability, agility, and vertical jump performance in young soccer players. Nevertheless, it remains important to determine whether, and to what extent, these associations are present in elite U-20 soccer players assessed within an applied professional-club testing environment.

Although both vertical jumps and rapid changes of direction require substantial lower-limb force production, they differ in their mechanical constraints. Vertical jumping involves rapid vertical impulse generation, whereas change-of-direction tasks require braking of the incoming momentum, reorientation of the body, and subsequent propulsive acceleration. The braking-to-propulsion transition involves lower-limb neuromuscular actions that may overlap with eccentric–concentric control during the countermovement jump. By comparison, average speed over an entire 30-m sprint combines initial acceleration with subsequent running and velocity-maintenance phases. These differences provided the biomechanical rationale for investigating whether jump variables were more closely associated with short directional-shuttle performance than with 30-m average sprint speed.

The aim of this study was to examine the degree of association among vertical jump performance, 30-m average sprint speed, and change-of-direction performance in elite U-20 soccer players. We hypothesized that vertical jump performance would be more consistently associated with change-of-direction performance than with 30-m average sprint speed.

## Materials and methods

### Study design and setting

This was a cross-sectional observational study based on a routine physical testing battery conducted in a professional soccer club in São Paulo, Brazil. The reporting of this study was guided by the Strengthening the Reporting of Observational Studies in Epidemiology (STROBE) statement. The testing sequence and principal findings are summarized in Fig 1.

**Fig 1:**
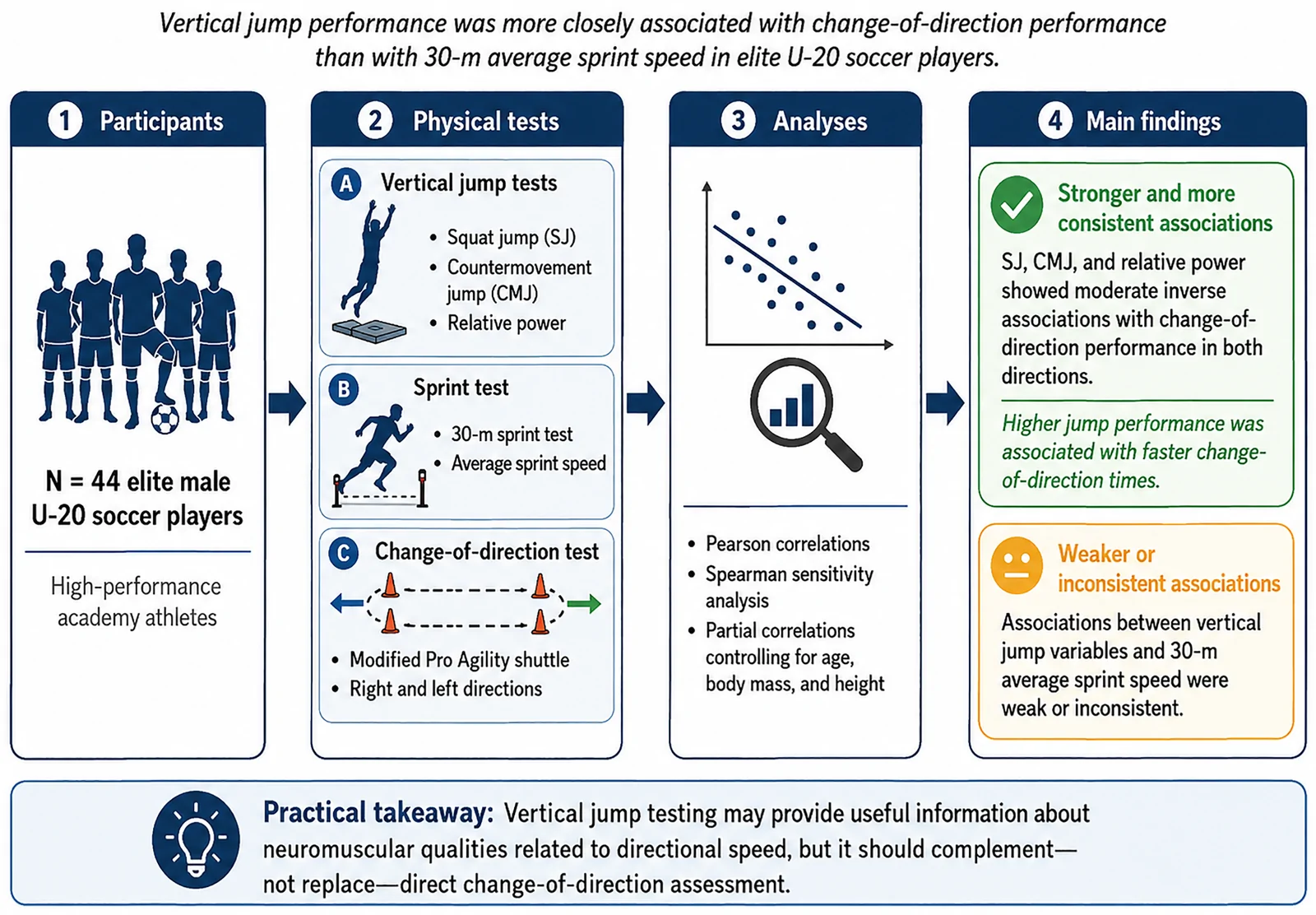
Graphical summary of study design, testing procedures, statistical analyses, and principal associations. Forty-four elite male U-20 soccer players completed squat jump, countermovement jump, 30-m sprint, and side-specific change-of-direction assessments. The figure summarizes the observed associations rather than implying causation.

### Ethics statement

This study was conducted in accordance with the Declaration of Helsinki and received approval from the Research Ethics Committee of Universidade Cidade de Sao Paulo under protocol number 7.872.376. Testing procedures were explained before data collection. Written informed consent was obtained from all participants; for athletes younger than 18 years, written informed consent was obtained from a parent or legal guardian and assent was obtained from the athlete. Recruitment began on January 1, 2026, and ended on June 1, 2026.

### Participants

Forty-four male elite under-20 soccer players participated in this study. Participant characteristics are presented in Table 1. The players competed at a high-performance level within a club involved in regional, national, continental, and intercontinental competitions. Regarding lower-limb dominance, 30 players were right-dominant and 14 were left-dominant. The most frequent playing positions were forwards (n = 13), midfielders (n = 9), fullbacks (n = 8), defensive midfielders (n = 7), and center backs (n = 7). To minimize the influence of fatigue, athletes were instructed to avoid strenuous physical activity for 48 hours before the assessments.

**Table 1:** Participant characteristics (N = 44).

Characteristic	Value
Age (years)	18.93 ± 0.87
Body mass (kg)	70.82 ± 5.80
Height (cm)	176.30 ± 7.10
Lower-limb dominance	
Right-dominant	30 (68.2%)
Left-dominant	14 (31.8%)
Playing position	
Forward	13 (29.5%)
Midfielder	9 (20.5%)
Defensive midfielder	7 (15.9%)
Fullback	8 (18.2%)
Center back	7 (15.9%)

*Values are mean ± SD or n (%).*

### Vertical jump testing

The athletes were familiar with the vertical jump tests because these assessments were part of the club's quarterly physical testing battery. Before data collection, athletes completed a 15-minute standardized warm-up supervised by the team's strength and conditioning staff. During the first 5 minutes, they performed dynamic stretching and self-selected preparatory exercises, provided that perceived exertion did not exceed 4 on the adapted Borg scale. During the next 5 minutes, they performed higher-intensity neuromuscular exercises, including squats and jumps. Approximately 5 minutes were then allocated for test-specific familiarization and standardization of instructions.

For the countermovement jump (CMJ), participants stood with their hands on their hips and jumped as high as possible after the evaluator’s verbal cue. Hip or knee flexion during flight was not permitted. Three maximal trials were performed, and the highest jump was retained. Testing was performed using a BIOMEC400 force platform (EMG System do Brasil Ltda., Brazil) measuring 600 × 400 mm and equipped with four force sensors and 16-bit acquisition. The vertical force signal was sampled at 1,000 Hz per channel, with a reported acquisition/filtering range of 0–50 Hz. Force-time data were visualized during acquisition and processed using the manufacturer’s EMGLab2 software, which identified take-off and landing phases automatically.

The squat jump (SJ) was assessed in the same testing session using the same platform and standardized verbal encouragement. Participants lowered into approximately 90° of knee flexion, maintained the position for 2 seconds, and then jumped maximally without a preceding countermovement. Three maximal trials were performed, with the best performance retained. Mean mechanical power was obtained from the force-platform analysis of each vertical jump; relative power was calculated by dividing platform-derived mean jump power (W) by the participant’s body mass (kg) and is expressed in W/kg. This normalization was used consistently in the manuscript and supporting tables.

### 30-m sprint testing

Linear sprint performance was evaluated using a 30-m sprint test, which was part of the club's routine physical assessment battery. This test was conducted 48 hours after the vertical jump assessments. Athletes completed a standardized warm-up supervised by the team's strength and conditioning coach, consisting of 5 minutes of dynamic stretching, 5 minutes of neuromuscular exercises, and 5 minutes of running coordination drills. Each athlete then performed two submaximal 30-m sprints before testing.

Each athlete performed two maximal 30-m sprints, and the trial with the shortest 30-m completion time was retained. Electronic timing gates (EMG System do Brasil, EMG Speed model) measured elapsed times at 5, 10, 20, and 30 m, with photoelectric reflectors positioned 2.5 m from their respective photocells. Athletes ran from a standing start following the evaluator’s audible cue and started when ready. Average speed over the full 30 m was calculated from the complete elapsed time as v_avg = (30 m / t_30m) × 3.6 and is reported in km/h. The gate arrangement also recorded intermediate cumulative splits, but only full-course average speed was used in the primary correlational analysis. This measure aggregates initial acceleration and later sprint phases; it should not be interpreted as directly measured maximal sprint velocity.

### Change-of-direction testing

The club’s routine change-of-direction assessment was a side-specific 5-m out-and-back shuttle recorded in the performance database as “Pro Agility right” and “Pro Agility left.” It was not the conventional 5-10-5 Pro Agility test or the standardized 5-0-5 protocol. Athletes started immediately in front of the electronic timing photocell in a slightly flexed-knee stance, with the hand on the intended travel side held close to the chest and the opposite arm directed downward. At the evaluator’s cue, they could initiate the task when ready. This was followed by travel 5 m to the designated right or left turning line, a deceleration and approximately 180° reversal, and a 5-m return through the same start/finish photocell, yielding approximately 10 m of total running per trial.

Each player completed two trials in each direction, in the sequence left–right–left–right, with the faster valid time retained separately for each side. An athlete had to place a foot on the ground beyond the 5-m turning marker; otherwise the trial was invalid and was repeated. The photoelectric timing system (EMG System do Brasil, EMG Speed model) was positioned 2.5 m from its reflector. Testing was preceded by a supervised 10-minute warm-up and a submaximal familiarization trial and was conducted approximately 48 hours after the 30-m sprint assessment. The two outcomes were therefore side-specific 10-m shuttle completion times (s), in which lower values indicate better performance.

### Statistical analysis

Statistical analyses were performed using IBM SPSS Statistics (IBM Corp., Armonk, NY, USA) and independently checked using Python scientific libraries. Continuous variables were described as mean ± standard deviation, and categorical variables were presented as absolute and relative frequencies. Normality was assessed using the Shapiro-Wilk test. Missing data were inspected before analysis. The uploaded raw dataset contained 44 complete athlete records for the variables included in the primary analyses.

Bivariate associations among SJ, CMJ, relative power, 30-m average sprint speed, and right- and left-sided change-of-direction performance were examined using Pearson product-moment correlation coefficients with 95% confidence intervals. Because CMJ and relative power deviated from normality, Spearman rank correlations were also calculated as a sensitivity analysis. To reduce the risk of type I error due to multiple testing, p-values were adjusted using the Benjamini-Hochberg false discovery rate procedure.

Correlation magnitudes were interpreted as trivial (<0.10), small (0.10–0.29), moderate (0.30–0.49), large (0.50–0.69), very large (0.70–0.89), and nearly perfect (0.90–0.99). Partial correlations were used to examine associations between vertical jump variables and performance outcomes after controlling for age, body mass, and height. Exploratory analyses examined potential differences according to lower-limb dominance and playing position. Because this was an applied observational study using an existing professional-club testing battery, no a priori sample size calculation was performed. Statistical significance was set at p < 0.05.

## Results

Participant characteristics are presented in Table 1.

Shapiro-Wilk tests indicated that CMJ and relative power deviated from normality, whereas SJ, 30-m average sprint speed, COD right, and COD left were approximately normally distributed. Pearson correlation analyses were used as the primary approach, and Spearman correlations were calculated as sensitivity analyses.

Pearson correlation analyses (Table 2) showed no statistically significant association between 30-m average sprint speed and SJ (r = 0.21, 95% CI = −0.09 to 0.48, p = 0.169), CMJ (r = 0.29, 95% CI = −0.01 to 0.54, p = 0.058; FDR-adjusted p = 0.078), or relative power (r = 0.28, 95% CI = −0.02 to 0.53, p = 0.071). Average sprint speed was also not significantly associated with COD right or COD left performance.

**Table 2:** Pearson correlations among vertical jump variables, 30-m average sprint speed, and change-of-direction performance (N = 44).

Variable	SJ	CMJ	Relative Power (W/kg)	30-m Average Sprint Speed (km/h)	COD Right (s)	COD Left (s)
SJ	1	0.812**	0.773**	0.211	−0.468**	−0.548**
CMJ	0.812**	1	0.878**	0.289	−0.487**	−0.522**
Relative Power (W/kg)	0.773**	0.878**	1	0.275	−0.425**	−0.558**
30-m Average Sprint Speed	0.211	0.289	0.275	1	−0.115	−0.252
COD Right (s)	−0.468**	−0.487**	−0.425**	−0.115	1	0.689**
COD Left (s)	−0.548**	−0.522**	−0.558**	−0.252	0.689**	1

Note. SJ = squat jump; CMJ = countermovement jump; COD = change of direction. * FDR-adjusted p < 0.05; ** FDR-adjusted p < 0.01. COD right and COD left refer to the side-specific 5-m out-and-back change-of-direction shuttle performed to each side.

Significant inverse associations were observed between vertical jump variables and change-of-direction performance. SJ was moderately associated with COD right (r = −0.47, 95% CI = −0.67 to −0.20, p = 0.001) and COD left (r = −0.55, 95% CI = −0.73 to −0.30, p = < 0.001). CMJ was also moderately associated with COD right (r = −0.49, 95% CI = −0.68 to −0.22, p = < 0.001) and COD left (r = −0.52, 95% CI = −0.71 to −0.27, p = < 0.001). Relative power showed similar inverse associations with COD right (r = −0.43, 95% CI = −0.64 to −0.15, p = 0.004) and COD left (r = −0.56, 95% CI = −0.73 to −0.31, p = < 0.001). These associations remained statistically significant after false discovery rate adjustment.

A large positive association was observed between COD right and COD left (r = 0.69, 95% CI = 0.49 to 0.82, p < 0.001), indicating that athletes who performed better in one direction also tended to perform better in the opposite direction, although the association was not perfect.

Partial correlation analyses controlling for age, body mass, and height showed the same overall pattern. Associations between SJ, CMJ, and relative power with COD right and COD left remained statistically significant (partial r range = −0.43 to −0.55, all p <= 0.005). In contrast, associations between vertical jump variables and 30-m average sprint speed were not statistically significant after adjustment.

Spearman rank correlations supported the Pearson results. Vertical jump variables remained significantly associated with COD right and COD left performance, whereas associations with 30-m average sprint speed were weak to small-to-moderate and not statistically significant after FDR adjustment.

Exploratory analyses showed no significant mean difference between COD right and COD left performance (mean difference = 0.01 s, p = 0.421). Lower-limb dominance was not significantly associated with SJ, CMJ, relative power, 30-m average sprint speed, or COD left performance. A trend was observed for COD right, with left-dominant players showing slower times than right-dominant players, although this did not reach statistical significance. Playing position was associated with 30-m average sprint speed (p = 0.029), with forwards showing higher average sprint speed than midfielders in post hoc testing. No significant positional differences were found for jump or change-of-direction variables.

## Discussion

The main finding of this study was that vertical jump performance was more consistently associated with change-of-direction performance than with 30-m average sprint speed in elite U-20 soccer players. SJ, CMJ, and relative power showed moderate inverse associations with right- and left-sided change-of-direction performance, whereas associations between vertical jump variables and 30-m average sprint speed were weak to small-to-moderate and did not remain statistically significant. This pattern remained stable after sensitivity analyses using Spearman correlations and after partial correlations controlling for age, body mass, and height.

These findings are partially consistent with previous observations in young soccer players reported by Köklü et al. [25], who found meaningful relationships among vertical jump performance, sprint ability, and agility performance. However, the present study adds a more specific applied interpretation: in elite U-20 soccer players, vertical jump performance appeared to be more closely related to a short directional speed task than to average speed across 30 m.

A plausible explanation is that change-of-direction performance depends heavily on rapid force production, braking capacity, transition mechanics, and re-acceleration. These qualities overlap with the neuromuscular demands involved in vertical jumping. In contrast, average speed over 30 m reflects a broader combination of acceleration profile, running mechanics, stride characteristics, and velocity maintenance. This broader composition may dilute its relationship with simple vertical jump measures.

The moderate magnitude of the observed associations should be interpreted carefully. The results do not indicate that vertical jump performance alone explains change-of-direction performance, nor do they imply that jump tests and change-of-direction tests are interchangeable. McFarland et al. [26] also reported associations between vertical jump tests and change-of-direction speed in collegiate soccer players, but the shared variance between these measures remains limited. More broadly, change-of-direction ability is recognized as multifactorial and dependent on strength, power, technical execution, braking strategy, body positioning, and task-specific mechanics [27].

The large, but not perfect, association between right- and left-sided change-of-direction performance also has practical relevance. Athletes who performed well in one direction tended to perform well in the opposite direction, but the association was clearly below unity and no systematic group-level directional bias was observed. Therefore, bilateral testing remains justified, because group means may conceal individual directional differences.

The exploratory analyses of lower-limb dominance suggest a similar interpretation. Lower-limb dominance was not significantly associated with most performance variables, although a non-significant trend was observed for right-sided change-of-direction performance. This finding should not be overinterpreted, because the study was not powered for subgroup comparisons. Nevertheless, it suggests that dominance may have task-specific relevance in side-dependent change-of-direction tests.

Playing position was associated only with 30-m average sprint speed. This is plausible because sprint demands and player profiling differ across soccer positions [6,18]. However, the positional analysis was exploratory and subgroup sizes were small. The finding should therefore be used to generate hypotheses rather than to support definitive position-specific conclusions.

Several limitations should be acknowledged. First, the sprint-related variable was average speed across the 30-m sprint, expressed in km/h, not directly measured maximal sprinting speed. Therefore, these findings should not be interpreted as evidence that vertical jump performance is weakly related to true maximal speed. Second, the change-of-direction task was a short, modified Pro Agility shuttle used in the club's testing battery. It should not be interpreted as a standard 5-0-5 test or as an isolated measure of change-of-direction skill. Third, the cross-sectional design only allows conclusions about association, not causation. It cannot be concluded that improving vertical jump performance will necessarily improve change-of-direction performance. Fourth, biological maturation was not assessed, and the study did not establish causality or an individual-level threshold for using jump tests as a proxy for directional performance.

Despite these limitations, the study has applied value. Vertical jump testing is fast, accessible, and commonly used in high-performance settings. The present findings suggest that SJ, CMJ, and relative power may provide useful information about neuromuscular qualities related to directional speed capacity in elite U-20 soccer players. However, coaches and practitioners should avoid using jump performance as a substitute for direct change-of-direction testing. The assessments should be considered complementary.

## Conclusions

In elite U-20 soccer players, vertical jump performance was more strongly and consistently associated with change-of-direction performance than with 30-m average sprint speed. SJ, CMJ, and relative power showed moderate inverse associations with right- and left-sided change-of-direction performance, whereas associations with average sprint speed were weak to small-to-moderate and not statistically significant after correction for multiple comparisons. These findings remained stable after controlling for age, body mass, and height and were supported by sensitivity analyses.

From an applied perspective, vertical jump testing may provide useful information about neuromuscular qualities related to directional speed performance. However, jump tests should complement rather than replace direct change-of-direction assessment. In addition, right- and left-sided performance were strongly related but not interchangeable, supporting bilateral assessment in soccer players.

## Supporting information

S1 File Supporting information tables. One DOCX file containing six tables: descriptive statistics and normality tests; Pearson correlations with 95% confidence intervals and FDR adjustment; Spearman correlations; partial correlations adjusted for age, body mass, and height; and exploratory analyses by lower-limb dominance and playing position.
(DOCX)
