# Supplementary material for: Vertical jump performance is more closely associated with change-of-direction performance than 30-m average sprint speed in elite U-20 soccer players
Source: PLoS One. 2026 Nov 12;21(11):e0355501. doi: 10.1371/journal.pone.0355501 (PMC13759038; doi:10.1371/journal.pone.0355501)
Supplement: Supplementary file 1 [file pone.0355501.s001.docx]

Supporting Information

Vertical jump performance is more closely associated with change-of-direction performance than 30-m average sprint speed in elite U-20 soccer players

**S1 Table. Descriptive statistics and Shapiro-Wilk normality tests for the main study variables (N = 44)**

| **Variable** | **Mean ± SD** | **Shapiro-Wilk W** | **p value** |
| --- | --- | --- | --- |
| SJ | 36.12 ± 4.82 | 0.958 | 0.112 |
| CMJ | 37.70 ± 5.23 | 0.943 | 0.030 |
| Relative Power (W/kg) | 29.11 ± 3.33 | 0.890 | <0.001 |
| 30-m Average Sprint Speed | 31.15 ± 1.09 | 0.971 | 0.335 |
| COD Right | 2.57 ± 0.13 | 0.990 | 0.972 |
| COD Left | 2.55 ± 0.17 | 0.975 | 0.457 |

*Note. SJ = squat jump; CMJ = countermovement jump; COD = change of direction. A p value < 0.05 indicates deviation from normality.*

**S2 Table. Pearson correlations with 95% confidence intervals and false discovery rate adjustment**

| **Variable 1** | **Variable 2** | **r** | **95% CI** | **p value** | **FDR-adjusted p** |
| --- | --- | --- | --- | --- | --- |
| SJ | CMJ | 0.812 | 0.678 to 0.893 | <0.001 | <0.001 |
| SJ | Relative Power (W/kg) | 0.773 | 0.618 to 0.870 | <0.001 | <0.001 |
| SJ | 30-m Average Sprint Speed | 0.211 | -0.091 to 0.478 | 0.169 | 0.181 |
| SJ | COD Right | -0.468 | -0.671 to -0.198 | 0.001 | 0.002 |
| SJ | COD Left | -0.548 | -0.726 to -0.299 | <0.001 | <0.001 |
| CMJ | Relative Power (W/kg) | 0.878 | 0.786 to 0.932 | <0.001 | <0.001 |
| CMJ | 30-m Average Sprint Speed | 0.289 | -0.009 to 0.539 | 0.058 | 0.078 |
| CMJ | COD Right | -0.487 | -0.685 to -0.222 | <0.001 | 0.002 |
| CMJ | COD Left | -0.522 | -0.709 to -0.266 | <0.001 | <0.001 |
| Relative Power (W/kg) | 30-m Average Sprint Speed | 0.275 | -0.024 to 0.529 | 0.071 | 0.088 |
| Relative Power (W/kg) | COD Right | -0.425 | -0.641 to -0.147 | 0.004 | 0.006 |
| Relative Power (W/kg) | COD Left | -0.558 | -0.733 to -0.313 | <0.001 | <0.001 |
| 30-m Average Sprint Speed | COD Right | -0.115 | -0.398 to 0.189 | 0.459 | 0.459 |
| 30-m Average Sprint Speed | COD Left | -0.252 | -0.511 to 0.048 | 0.099 | 0.114 |
| COD Right | COD Left | 0.689 | 0.493 to 0.818 | <0.001 | <0.001 |

*Note. FDR = Benjamini-Hochberg false discovery rate procedure.*

**S3 Table. Spearman rank correlations as sensitivity analysis**

| **Variable 1** | **Variable 2** | **Spearman's rho** | **p value** | **FDR-adjusted p** |
| --- | --- | --- | --- | --- |
| SJ | CMJ | 0.707 | <0.001 | <0.001 |
| SJ | Relative Power (W/kg) | 0.684 | <0.001 | <0.001 |
| SJ | 30-m Average Sprint Speed | 0.201 | 0.192 | 0.221 |
| SJ | COD Right | -0.448 | 0.002 | 0.004 |
| SJ | COD Left | -0.482 | <0.001 | 0.002 |
| CMJ | Relative Power (W/kg) | 0.826 | <0.001 | <0.001 |
| CMJ | 30-m Average Sprint Speed | 0.263 | 0.084 | 0.115 |
| CMJ | COD Right | -0.519 | <0.001 | <0.001 |
| CMJ | COD Left | -0.486 | <0.001 | 0.002 |
| Relative Power (W/kg) | 30-m Average Sprint Speed | 0.228 | 0.137 | 0.171 |
| Relative Power (W/kg) | COD Right | -0.438 | 0.003 | 0.004 |
| Relative Power (W/kg) | COD Left | -0.468 | 0.001 | 0.003 |
| 30-m Average Sprint Speed | COD Right | -0.112 | 0.469 | 0.469 |
| 30-m Average Sprint Speed | COD Left | -0.171 | 0.268 | 0.287 |
| COD Right | COD Left | 0.686 | <0.001 | <0.001 |

**S4 Table. Partial correlations controlling for age, body mass, and height**

| **Variable 1** | **Variable 2** | **Partial r** | **95% CI** | **p value** |
| --- | --- | --- | --- | --- |
| SJ | 30-m Average Sprint Speed | 0.164 | -0.152 to 0.449 | 0.307 |
| SJ | COD Right | -0.458 | -0.671 to -0.175 | 0.003 |
| SJ | COD Left | -0.546 | -0.731 to -0.286 | <0.001 |
| CMJ | 30-m Average Sprint Speed | 0.299 | -0.009 to 0.556 | 0.057 |
| CMJ | COD Right | -0.497 | -0.698 to -0.224 | <0.001 |
| CMJ | COD Left | -0.517 | -0.712 to -0.249 | <0.001 |
| Relative Power (W/kg) | 30-m Average Sprint Speed | 0.305 | -0.003 to 0.560 | 0.052 |
| Relative Power (W/kg) | COD Right | -0.429 | -0.651 to -0.140 | 0.005 |
| Relative Power (W/kg) | COD Left | -0.547 | -0.731 to -0.288 | <0.001 |

**S5 Table. Exploratory analysis according to lower-limb dominance**

| **Variable** | **Right-dominant (n = 30)** | **Left-dominant (n = 14)** | **p value** |
| --- | --- | --- | --- |
| SJ | 36.10 ± 4.59 | 36.15 ± 5.46 | 0.978 |
| CMJ | 37.88 ± 4.60 | 37.30 ± 6.57 | 0.768 |
| Relative Power (W/kg) | 28.89 ± 3.21 | 29.59 ± 3.63 | 0.546 |
| 30-m Average Sprint Speed | 31.18 ± 1.14 | 31.09 ± 1.03 | 0.779 |
| COD Right | 2.54 ± 0.13 | 2.62 ± 0.12 | 0.055 |
| COD Left | 2.54 ± 0.16 | 2.58 ± 0.18 | 0.567 |

*Note. Values are mean ± SD. Between-group p values were calculated using Welch’s t tests.*

**S6 Table. Exploratory analysis according to playing position**

| **Variable** | **Forward (n = 13)** | **Midfielder (n = 9)** | **Defensive midfielder (n = 7)** | **Fullback (n = 8)** | **Center back (n = 7)** | **p value** |
| --- | --- | --- | --- | --- | --- | --- |
| SJ | 37.98 ± 5.46 | 35.30 ± 6.11 | 36.50 ± 5.48 | 35.41 ± 2.42 | 34.13 ± 2.48 | 0.476 |
| CMJ | 39.88 ± 6.04 | 38.48 ± 5.58 | 36.49 ± 5.59 | 35.79 ± 3.99 | 36.03 ± 3.28 | 0.337 |
| Relative Power (W/kg) | 30.33 ± 4.34 | 29.68 ± 3.22 | 28.84 ± 3.60 | 28.30 ± 1.96 | 27.31 ± 1.42 | 0.343 |
| 30-m Average Sprint Speed | 31.66 ± 0.91 | 30.36 ± 0.77 | 31.30 ± 1.06 | 30.71 ± 0.85 | 31.59 ± 1.45 | 0.029 |
| COD Right | 2.51 ± 0.16 | 2.58 ± 0.08 | 2.56 ± 0.09 | 2.59 ± 0.13 | 2.66 ± 0.12 | 0.186 |
| COD Left | 2.49 ± 0.20 | 2.55 ± 0.14 | 2.52 ± 0.13 | 2.62 ± 0.16 | 2.64 ± 0.16 | 0.290 |

*Note. Values are mean ± SD. One-way ANOVA identified a significant positional effect only for 30-m average sprint speed. Tukey post hoc testing indicated that forwards showed higher average sprint speed than midfielders (adjusted p = 0.035).*
