# Supplementary material for: Vertical jump performance is more closely associated with change-of-direction performance than 30-m average sprint speed in elite U-20 soccer players
Source: PLoS One. 2026 Nov 12;21(11):e0355501. doi: 10.1371/journal.pone.0355501 (PMC13759038; doi:10.1371/journal.pone.0355501)
Supplement: Supplementary file 2 [file pone.0355501.s003.docx]

Response to Reviewers

PLOS ONE — Manuscript PONE-D-26-29543

Vertical jump performance is more closely associated with change-of-direction performance than 30-m average sprint speed in elite U-20 soccer players

Dear Dr. Cè and Reviewer,

We thank the Academic Editor and the reviewer for their constructive evaluation. We have revised the manuscript to improve methodological reproducibility, clarify the interpretation of average 30-m sprint speed, align figure captions and ethics reporting with journal requirements, and complete authorship declarations. We retained the submitted analytic sample of 44 athletes and the original correlation analyses, confidence intervals, and false discovery rate corrections; the reviewer did not request a change to the underlying analyses. Below we address the points individually.

## Academic Editor — overall assessment

**Comment.** The reviewer raises several minor points requiring revision.

**Response.** We have addressed methodological reporting, declaration completeness, presentation of the figure caption, and the interpretation of sprint speed. Textual changes are identified in the accompanying marked-up manuscript.

## Journal requirement 1 — formatting

**Comment.** Ensure manuscript style and file naming meet PLOS ONE requirements.

**Response.** The revised manuscript uses a consistent title page, section headings, original tables located within the manuscript, and a separate supporting-information document. The figure file remains separate and its corresponding caption appears in the text.

## Journal requirement 2 — ethics location

**Comment.** The ethics statement must appear only in the Methods section.

**Response.** We removed the ethics approval and consent paragraphs previously duplicated in the front matter. The complete ethics and consent description is now confined to the Materials and methods section under “Ethics statement.”

## Journal requirement 3 — figure caption

**Comment.** Include a separate caption for each figure in the manuscript.

**Response.** We inserted an explicit reference to Fig 1 and a separate descriptive caption immediately following its first mention in the Study design and setting subsection. The scientific content of the separately submitted figure did not require revision.

## Reviewer — platform frequency and equipment

**Comment.** Report force-platform sampling frequency and sufficient equipment specifications.

**Response.** The Vertical jump testing subsection now specifies the BIOMEC400 force platform (EMG System do Brasil), its 600 × 400 mm dimensions, four sensors, 16-bit acquisition, sampling frequency of 1,000 Hz per channel, the reported 0–50 Hz acquisition/filtering range, and manufacturer EMGLab2 software. These details came from the measurement-team documentation.

## Reviewer A — abstract sprint speed

**Comment.** Clarify that 30-m average sprint speed derives from overall time and is distinct from maximal linear sprint speed.

**Response.** We specify in the Abstract that average 30-m speed was derived from the total sprint time. Materials and methods now gives the explicit formula v_avg = (30 m/t_30m) × 3.6 and states that this quantity is not directly measured maximal sprint velocity.

## Reviewer B — biomechanical rationale

**Comment.** Expand the rationale for a stronger association between jumping and COD than full-course average sprint speed.

**Response.** We added an introductory explanation of the partially shared lower-limb demands of vertical impulse generation, braking, rapid momentum redirection, and subsequent propulsion during directional shuttles; we contrast these with the heterogeneous acceleration and velocity-maintenance components contributing to 30-m average sprint speed. The interpretation remains associational rather than causal.

## Reviewer C1 — COD protocol

**Comment.** Specify exact change-of-direction course length, turns and starting posture.

**Response.** We describe a short 5-m out-and-back course (approximately 10 m total), start/finish photocell arrangement, 180-degree reversal, slightly flexed-knee stance, initial arm position, trial sequence (left–right–left–right), a foot-contact validity criterion, best-trial selection, warm-up, and device/reflector geometry. We clarify that the club label “Pro Agility” does not imply a conventional 5-10-5 or standardized 5-0-5 protocol.

## Reviewer C2 — relative power

**Comment.** Explain the calculation of relative jump power from the platform output.

**Response.** We specify that relative power was obtained as force-platform-derived mean vertical-jump power in watts divided by athlete body mass in kilograms, reported as W/kg. To remove an ambiguous original abbreviation, we also harmonized W/BW to W/kg in the correlation and supporting-information tables; no values were changed.

## Reviewer C3 — sprint gates

**Comment.** Confirm intermediate timing gates and explain full-course speed.

**Response.** We clarify that electronic timing gates recorded 5, 10, 20 and 30 m cumulative times, with 2.5-m photocell-to-reflector spacing. The principal outcome remained total-distance average speed from 30-m completion time and consequently conflates initial acceleration with later running phases.

## Reviewer D — CRediT roles

**Comment.** Complete Author Contributions and remove placeholders.

**Response.** The revised Author contributions section provides named CRediT roles for all eleven authors, based on the completed contribution list in the submitted title page. All incomplete placeholders were removed and the duplicate front-matter contribution statement was eliminated.

## Reviewer E — discussion/subgroups

**Comment.** Keep interpretations of moderate associations and exploratory dominance/position comparisons cautious.

**Response.** We retained the qualifying discussion that jump tests complement but cannot replace direct COD testing, and that position/dominance analyses are exploratory and underpowered. No additional inferential claims were added.

## Additional editorial clean-up — declarations and supporting materials

**Comment.** Remove unfinished author-facing instructions and ensure accurate data statements.

**Response.** We deleted editing instructions in the Acknowledgments, Funding and Competing interests sections. The complete supplementary analyses (S1–S6) retain the original 44-athlete analysis and unchanged numerical estimates. Figure 1 retains its original graphical presentation. We replaced the incorrect Figshare link previously repeated in the title page with the alternative dataset DOI already identified in the original manuscript/cover letter.

We appreciate the opportunity to submit the revised version of this manuscript for further consideration.

Sincerely,
Robson Dias Scoz, PhD
Corresponding Author, on behalf of the coauthors
