# Supplementary material for: Vertical jump performance is more closely associated with change-of-direction performance than 30-m average sprint speed in elite U-20 soccer players
Source: PLoS One. 2026 Nov 12;21(11):e0355501. doi: 10.1371/journal.pone.0355501 (PMC13759038; doi:10.1371/journal.pone.0355501)
Supplement: Peer review history

# Peer review: Decision Letter 0

**DOI:** 10.1371/journal.pone.0355501.r001

**Authors:** Emiliano Cè

7 Oct 2026

Dear Dr. Scoz,

Thank you for submitting your manuscript to PLOS One. After careful consideration, we feel that it has merit but does not fully meet PLOS One’s publication criteria as it currently stands. Therefore, we invite you to submit a revised version of the manuscript that addresses the points raised during the review process.

==============================

**ACADEMIC EDITOR:** plosone@plos.org. When you're ready to submit your revision, log on to https://www.editorialmanager.com/pone/ and select the 'Submissions Needing Revision' folder to locate your manuscript file.

- A letter that responds to each point raised by the academic editor and reviewer(s). You should upload this letter as a separate file labeled 'Response to Reviewers'.
- A marked-up copy of your manuscript that highlights changes made to the original version. You should upload this as a separate file labeled 'Revised Manuscript with Track Changes'.
- An unmarked version of your revised paper without tracked changes. You should upload this as a separate file labeled 'Manuscript'.

If you would like to make changes to your financial disclosure, please include your updated statement in your cover letter. Guidelines for resubmitting your figure files are available below the reviewer comments at the end of this letter.

If applicable, we recommend that you deposit your laboratory protocols in protocols.io to enhance the reproducibility of your results. Protocols.io assigns your protocol its own identifier (DOI) so that it can be cited independently in the future. For instructions see: https://journals.plos.org/plosone/s/submission-guidelines#loc-laboratory-protocols. Additionally, PLOS One offers an option for publishing peer-reviewed Lab Protocol articles, which describe protocols hosted on protocols.io. Read more information on sharing protocols at https://plos.org/protocols?utm_medium=editorial-email&utm_source=authorletters&utm_campaign=protocols.

As the corresponding author, your ORCID iD is verified in the submission system and will appear in the published article. PLOS supports the use of ORCID, and we encourage all coauthors to register for an ORCID iD and use it as well. Please encourage your coauthors to verify their ORCID iD within the submission system before final acceptance, as unverified ORCID iDs will not appear in the published article. *Only*  the individual author can complete the verification step; PLOS staff *cannot*  verify ORCID iDs on behalf of authors.

We look forward to receiving your revised manuscript.

Kind regards,

Emiliano Cè, Ph.D.

Academic Editor

PLOS One

Journal requirements:

When submitting your revision, we need you to address these additional requirements.

1. Please ensure that your manuscript meets PLOS ONE's style requirements, including those for file naming. The PLOS ONE style templates can be found at

https://journals.plos.org/plosone/s/file?id=wjVg/PLOSOne_formatting_sample_main_body.pdf and

https://journals.plos.org/plosone/s/file?id=ba62/PLOSOne_formatting_sample_title_authors_affiliations.pdf

2. Your ethics statement should only appear in the Methods section of your manuscript. If your ethics statement is written in any section besides the Methods, please delete it from any other section.

3. Please include a separate caption for each figure in your manuscript.

If the reviewer comments include a recommendation to cite specific previously published works, please review and evaluate these publications to determine whether they are relevant and should be cited. There is no requirement to cite these works unless the editor has indicated otherwise.

Please review your reference list to ensure that it is complete and correct. If you have cited papers that have been retracted, please include the rationale for doing so in the manuscript text, or remove these references and replace them with relevant current references. Any changes to the reference list should be mentioned in the rebuttal letter that accompanies your revised manuscript. If you need to cite a retracted article, indicate the article’s retracted status in the References list and also include a citation and full reference for the retraction notice.

[Note: HTML markup is below. Please do not edit.]

Reviewers' comments:

Reviewer's Responses to Questions

**Comments to the Author**

1. Is the manuscript technically sound, and do the data support the conclusions?

Reviewer #1: Partly

**********

2. Has the statistical analysis been performed appropriately and rigorously?

Reviewer #1: Yes

**********

3. Have the authors made all data underlying the findings in their manuscript fully available?

The PLOS Data policy

Reviewer #1: Yes

**********

4. Is the manuscript presented in an intelligible fashion and written in standard English?

Reviewer #1: Yes

**********

Reviewer #1: Rigorous Overall Design and Statistical Analyses: The study employs a sound cross-sectional observational design using appropriate correlation analyses (Pearson and Spearman), controlling for confounding variables (age, height, and body mass), and applying Benjamini-Hochberg False Discovery Rate (FDR) adjustments to mitigate Type I error risks. The presented data generally support the core conclusion regarding moderate inverse associations between vertical jump metrics and change-of-direction performance.

Insufficient Description of Protocols and Equipment: Essential methodological details required for full replication are missing, such as the force platform's sampling frequency (in Hz) and whether intermediate timing gates were used during the 30-m sprint test or if only a single end-gate was utilized.

Incomplete Declarations: The manuscript contains placeholder text in the Author Contributions section (e.g., [to be completed]), which must be fully specified according to CRediT taxonomy to establish complete technical and administrative soundness prior to publication.

1. General Evaluation & Summary This study examines the relationship between vertical jump performance (SJ, CMJ, relative power), 30-m average sprint speed, and side-specific change-of-direction (COD) performance in a cohort of 44 elite U-20 male soccer players from a professional Brazilian club. The authors employed appropriate bivariate correlations (Pearson and Spearman), controlling for multiple comparisons (Benjamini-Hochberg FDR) and confounding variables (age, body mass, height). The primary finding is that vertical jump measures are moderately and inversely correlated with COD times to both sides ($r = -0.43$ to $-0.56$), while associations with 30-m average sprint speed were weaker ($r = 0.21$ to $0.29$) and not statistically significant after FDR adjustment. Overall, the manuscript is well-structured, methodologically sound, and addresses a practical question in sports conditioning. However, minor issues regarding terminology, methodology reporting, and formatting in the declaration sections need to be addressed before publication.

Major & Minor Revisions RequestedA. Title & Abstract Title: Accurate and reflective of the findings. Abstract (Line 63): Clarify that "average speed" across 30 m was derived from overall time, as this is distinct from maximal linear sprinting velocity (Vmax). B. Introduction Lines 100–103: The rationale for why jumping would correlate better with COD than 30-m sprint speed is brief. Please expand slightly on the neuromuscular biomechanics shared between vertical impulse and horizontal decelerations/re-accelerations in COD. C. Materials and Methods COD Task Terminology (Lines 160–164): The authors refer to the COD task as a "modified Pro Agility shuttle". Please detail the exact distance, turns, and starting posture so that the protocol is fully reproducible by independent researchers. Force Platform & Jump Protocols (Lines 139–145): Provide more specifications for the force platform (e.g., sampling frequency in Hz). State how relative power was calculated from force-time data. Sprint Speed Calculation (Lines 157–158): Clarify if timing gates were placed at intermediate splits (e.g., 10m, 20m) or only at 30m. Measuring average speed across 30 m mixes the acceleration phase with top-speed maintenance. This limitation is noted in the Discussion, but should be explicitly mentioned in the Methods. D. Results Table 2: Data presentation is clean and well-annotated with FDR markers. Incomplete Author Contributions Section (Lines 318–322): In the main body text, the Author contributions section contains place-holders like [to be completed]. Ensure that all author initials and their exact roles (using CRediT taxonomy) are completely filled in prior to final acceptance. E. Discussion & Practical Applications Lines 251–254: The discussion correctly acknowledges the moderate strength of the correlations (r^2 \approx 18-31\%), emphasizing that vertical jump tests cannot replace direct COD testing. This nuanced interpretation is commendable. Position & Dominance Subgroups (Lines 268–277): The discussion appropriately notes that subgroup analysis was exploratory and underpowered. Keep these interpretations cautious. 4. Conclusion for Editor Decision: Accept with Minor Revisions. Justification: The scientific approach is solid, statistical adjustments are robust, ethical requirements are fulfilled, and raw data is accessible. Fixing the placeholders in the Author Contributions section and clarifying the exact COD testing distance/equipment parameters will make the paper ready for publication in PLOS ONE.

**********

what does this mean?). If published, this will include your full peer review and any attached files.

If you choose “no”, your identity will remain anonymous but your review may still be made public.

**Do you want your identity to be public for this peer review?**  For information about this choice, including consent withdrawal, please see our Privacy Policy

Reviewer #1: No

**********

[NOTE: If reviewer comments were submitted as an attachment file, they will be attached to this email and accessible via the submission site. Please log into your account, locate the manuscript record, and check for the action link "View Attachments". If this link does not appear, there are no attachment files.]

To ensure your figures meet our technical requirements, please review our figure guidelines: https://journals.plos.org/plosone/s/figures

You may also use PLOS’s free figure tool, NAAS, to help you prepare publication quality figures: https://journals.plos.org/plosone/s/figures#loc-tools-for-figure-preparation.

NAAS will assess whether your figures meet our technical requirements by comparing each figure against our figure specifications.

# Author comment: Author response to Decision Letter 1

**DOI:** 10.1371/journal.pone.0355501.r002

15 Oct 2026

Response to Reviewers

PLOS ONE — Manuscript PONE-D-26-29543

Vertical jump performance is more closely associated with change-of-direction performance than 30-m average sprint speed in elite U-20 soccer players

Dear Dr. Cè and Reviewer,

We thank the Academic Editor and the reviewer for their constructive evaluation. We have revised the manuscript to improve methodological reproducibility, clarify the interpretation of average 30-m sprint speed, align figure captions and ethics reporting with journal requirements, and complete authorship declarations. We retained the submitted analytic sample of 44 athletes and the original correlation analyses, confidence intervals, and false discovery rate corrections; the reviewer did not request a change to the underlying analyses. Below we address the points individually.

Academic Editor — overall assessment

Comment. The reviewer raises several minor points requiring revision.

Response. We have addressed methodological reporting, declaration completeness, presentation of the figure caption, and the interpretation of sprint speed. Textual changes are identified in the accompanying marked-up manuscript.

Journal requirement 1 — formatting

Comment. Ensure manuscript style and file naming meet PLOS ONE requirements.

Response. The revised manuscript uses a consistent title page, section headings, original tables located within the manuscript, and a separate supporting-information document. The figure file remains separate and its corresponding caption appears in the text.

Journal requirement 2 — ethics location

Comment. The ethics statement must appear only in the Methods section.

Response. We removed the ethics approval and consent paragraphs previously duplicated in the front matter. The complete ethics and consent description is now confined to the Materials and methods section under “Ethics statement.”

Journal requirement 3 — figure caption

Comment. Include a separate caption for each figure in the manuscript.

Response. We inserted an explicit reference to Fig 1 and a separate descriptive caption immediately following its first mention in the Study design and setting subsection. The scientific content of the separately submitted figure did not require revision.

Reviewer — platform frequency and equipment

Comment. Report force-platform sampling frequency and sufficient equipment specifications.

Response. The Vertical jump testing subsection now specifies the BIOMEC400 force platform (EMG System do Brasil), its 600 × 400 mm dimensions, four sensors, 16-bit acquisition, sampling frequency of 1,000 Hz per channel, the reported 0–50 Hz acquisition/filtering range, and manufacturer EMGLab2 software. These details came from the measurement-team documentation.

Reviewer A — abstract sprint speed

Comment. Clarify that 30-m average sprint speed derives from overall time and is distinct from maximal linear sprint speed.

Response. We specify in the Abstract that average 30-m speed was derived from the total sprint time. Materials and methods now gives the explicit formula v_avg = (30 m/t_30m) × 3.6 and states that this quantity is not directly measured maximal sprint velocity.

Reviewer B — biomechanical rationale

Comment. Expand the rationale for a stronger association between jumping and COD than full-course average sprint speed.

Response. We added an introductory explanation of the partially shared lower-limb demands of vertical impulse generation, braking, rapid momentum redirection, and subsequent propulsion during directional shuttles; we contrast these with the heterogeneous acceleration and velocity-maintenance components contributing to 30-m average sprint speed. The interpretation remains associational rather than causal.

Reviewer C1 — COD protocol

Comment. Specify exact change-of-direction course length, turns and starting posture.

Response. We describe a short 5-m out-and-back course (approximately 10 m total), start/finish photocell arrangement, 180-degree reversal, slightly flexed-knee stance, initial arm position, trial sequence (left–right–left–right), a foot-contact validity criterion, best-trial selection, warm-up, and device/reflector geometry. We clarify that the club label “Pro Agility” does not imply a conventional 5-10-5 or standardized 5-0-5 protocol.

Reviewer C2 — relative power

Comment. Explain the calculation of relative jump power from the platform output.

Response. We specify that relative power was obtained as force-platform-derived mean vertical-jump power in watts divided by athlete body mass in kilograms, reported as W/kg. To remove an ambiguous original abbreviation, we also harmonized W/BW to W/kg in the correlation and supporting-information tables; no values were changed.

Reviewer C3 — sprint gates

Comment. Confirm intermediate timing gates and explain full-course speed.

Response. We clarify that electronic timing gates recorded 5, 10, 20 and 30 m cumulative times, with 2.5-m photocell-to-reflector spacing. The principal outcome remained total-distance average speed from 30-m completion time and consequently conflates initial acceleration with later running phases.

Reviewer D — CRediT roles

Comment. Complete Author Contributions and remove placeholders.

Response. The revised Author contributions section provides named CRediT roles for all eleven authors, based on the completed contribution list in the submitted title page. All incomplete placeholders were removed and the duplicate front-matter contribution statement was eliminated.

Reviewer E — discussion/subgroups

Comment. Keep interpretations of moderate associations and exploratory dominance/position comparisons cautious.

Response. We retained the qualifying discussion that jump tests complement but cannot replace direct COD testing, and that position/dominance analyses are exploratory and underpowered. No additional inferential claims were added.

Additional editorial clean-up — declarations and supporting materials

Comment. Remove unfinished author-facing instructions and ensure accurate data statements.

Response. We deleted editing instructions in the Acknowledgments, Funding and Competing interests sections. The complete supplementary analyses (S1–S6) retain the original 44-athlete analysis and unchanged numerical estimates. Figure 1 retains its original graphical presentation. We replaced the incorrect Figshare link previously repeated in the title page with the alternative dataset DOI already identified in the original manuscript/cover letter.

We appreciate the opportunity to submit the revised version of this manuscript for further consideration.

Sincerely,

Robson Dias Scoz, PhD

Corresponding Author, on behalf of the coauthors

Attachment Submitted filename: PONE-D-26-29543_Response_to_Reviewers.docx

# Peer review: Decision Letter 1

**DOI:** 10.1371/journal.pone.0355501.r003

**Authors:** Emiliano Cè

28 Oct 2026

Vertical jump performance is more closely associated with change-of-direction performance than 30-m average sprint speed in elite U-20 soccer players

PONE-D-26-29543R1

Dear Dr. Scoz,

We’re pleased to inform you that your manuscript has been judged scientifically suitable for publication and will be formally accepted for publication once it meets all outstanding technical requirements.

Within one week, you’ll receive an e-mail detailing the required amendments. When these have been addressed, you’ll receive a formal acceptance letter and your manuscript will be scheduled for publication.

An invoice will be generated when your article is formally accepted. Please note, if your institution has a publishing partnership with PLOS and your article meets the relevant criteria, all or part of your publication costs will be covered. Please make sure your user information is up-to-date by logging into Editorial Manager at Editorial Manager® and clicking the ‘Update My Information' link at the top of the page. For questions related to billing, please contact billing support.

If your institution or institutions have a press office, please notify them about your upcoming paper to help maximize its impact. If they’ll be preparing press materials, please inform our press team as soon as possible -- no later than 48 hours after receiving the formal acceptance. Your manuscript will remain under strict press embargo until 2 pm Eastern Time on the date of publication. For more information, please contact onepress@plos.org.

Kind regards,

Emiliano Cè, Ph.D.

Academic Editor

PLOS One

Additional Editor Comments (optional):

Reviewers' comments:

Reviewer's Responses to Questions

**Comments to the Author**

Reviewer #1: All comments have been addressed

**********

2. Is the manuscript technically sound, and do the data support the conclusions?

Reviewer #1: Yes

**********

3. Has the statistical analysis been performed appropriately and rigorously?

Reviewer #1: Yes

**********

4. Have the authors made all data underlying the findings in their manuscript fully available?

The PLOS Data policy

Reviewer #1: Yes

**********

5. Is the manuscript presented in an intelligible fashion and written in standard English?

Reviewer #1: Yes

**********

Reviewer #1: Reviewer Comments:

The authors have thoroughly and meticulously addressed all comments, methodological queries, and structural suggestions raised during the previous round of review. Specifically :Methodology & Equipment: The force platform specifications (BIOMEC400, sampling frequency, dimensions, and filtering range) and relative power normalization (W/kg) are now reported with high precision. Sprint & COD Protocols: The definition and formula for 30-m average sprint speed have been explicitly clarified, ensuring it is properly distinguished from maximal linear speed. Furthermore, the specific mechanics and setup of the modified Pro Agility change-of-direction shuttle have been transparently detailed. Statistical Rigor & Ethics: The use of false discovery rate (FDR) adjustments, sensitivity analyses (Spearman and partial correlations controlling for age, height, and body mass), and ethical compliance statements under the Materials and Methods section significantly strengthen the manuscript's validity. The manuscript is now methodologically sound, transparent, and provides valuable applied insights into the neuromuscular profiling of elite youth soccer players. Therefore, I recommend the manuscript for publication in its current form.

**********

what does this mean?). If published, this will include your full peer review and any attached files.

If you choose “no”, your identity will remain anonymous but your review may still be made public.

**Do you want your identity to be public for this peer review?**  For information about this choice, including consent withdrawal, please see our Privacy Policy

Reviewer #1: No

**********

# Editor report: Acceptance letter

**DOI:** 10.1371/journal.pone.0355501.r004

**Authors:** Emiliano Cè

PONE-D-26-29543R1

PLOS One

Dear Dr. Scoz,

I'm pleased to inform you that your manuscript has been deemed suitable for publication in PLOS One. Congratulations! Your manuscript is now being handed over to our production team.

At this stage, our production department will prepare your paper for publication. This includes ensuring the following:

* All references, tables, and figures are properly cited

* All relevant supporting information is included in the manuscript submission,

* There are no issues that prevent the paper from being properly typeset

You will receive further instructions from the production team, including instructions on how to review your proof when it is ready. Please keep in mind that we are working through a large volume of accepted articles, so please give us a few days to review your paper and let you know the next and final steps.

Lastly, if your institution or institutions have a press office, please let them know about your upcoming paper now to help maximize its impact. If they'll be preparing press materials, please inform our press team within the next 48 hours. Your manuscript will remain under strict press embargo until 2 pm Eastern Time on the date of publication. For more information, please contact onepress@plos.org.

You will receive an invoice from PLOS for your publication fee after your manuscript has reached the completed accept phase. If you receive an email requesting payment before acceptance or for any other service, this may be a phishing scheme. Learn how to identify phishing emails and protect your accounts at https://explore.plos.org/phishing.

If we can help with anything else, please email us at customercare@plos.org.

Thank you for submitting your work to PLOS One and supporting open access.

Kind regards,

PLOS One Editorial Office Staff

on behalf of

Prof. Emiliano Cè

Academic Editor

PLOS One
